# The complete chloroplast genome of *Melicope pteleifolia* (*Rutaceae*), a traditional medicinal plant in Southeast China

**DOI:** 10.1080/23802359.2020.1837026

**Published:** 2021-01-14

**Authors:** Jingwei Yu, Luxi Chen, Jianwen Mao, Xiaobao Jin, Juan Shen

**Affiliations:** a School of Life Science and Biopharmaceutics, Guangdong Pharmaceutical University, Guangzhou, PR China; b Guangdong Key Laboratory of Pharmaceutical Bioactive Substances, Guangdong Pharmaceutical University, Guangzhou, PR China

**Keywords:** Chloroplast genome, next-generation sequencing, *Melicope pterleifolia*, phylogenetic relationships analysis

## Abstract

*Meclicope pteleifolia* is a traditional medicinal herb and edible plant in Southeast China. Here, we report the complete chloroplast genome of *M. pteleifolia*. The chloroplast genome is 159,012 bp in length with 38.33% GC content, containing a small single-copy (SSC) region (18,609 bp), a large single-copy (LSC) region (851 bp), and a pair of inverted repeats (IRs: 27,640 bp each). A total of 131 genes were predicted, including 84 protein-coding genes, 8 ribosomal RNA genes, 37 tRNA genes, and 2 pseudogenes. Phylogenetic analysis based on chloroplast genomes of 17 plant species shows that *M. pteleifolia* is closest to *Zanthoxylum* and *Casimiroa*. These complete chloroplast genomes can be subsequently used for researches of Rutaceae.

*Melicope pterleifolia* is a perennial herbaceous shrub or tree that belongs to Rosaceae and is widely distributed in southern China and Southeast Asian. Its stems, roots, and barks are used in Chinese traditional medicine with the common name ‘sanyaku’, and can act as anti-inflammatory, antipyretic agent to treat hemorrhoids, eczema, dermatitis, abscesses, and trauma. On the other hand, *M. pterleifolia* is also edible, its fresh leaves are a common vegetable in Malaysia, meanwhile, these leaves are the main ingredient of herbal tea, a popular healthy drink in Guangdong province, China (Zhang et al. 2012). In this article, we assembled the complete chloroplast genome of *M. pterleifolia* and explored the phylogenetic relationship with other species in Rutaceae family.

Fresh and young leaves of *M. pterleifolia* were collected from arboretum in South China Agriculture University, Guangzhou, Guangdong Province, China (N23°16′29.93″, E113°35′90.71″), and the voucher specimen was deposited in the Herbarium of South China Agricultural University (CANT) under the accession number 440523-190806-022. The total genomic DNA was extracted by using a modified CTAB method (Doyle and Doyle 1987), and library consisting of an insert size of 300 bp was constructed using TruSeq DNA Sample Prep Kit (Illumina), and sequencing was carried out on an Illumina HiSeq Nova platform (Illumina, USA). About 6 Gb raw data of paired-end reads (150 bp) were produced and further assembled using GetOrganelle (Jin et al. 2018). The cp genome was annotated using Geseq (Tillich et al. 2017) and then manually checked by comparison against the complete cp genome of *Zanthoxylum piperitum* (Genbank accession number: KT153018). The complete chloroplast genome of *M. pterleifolia* was submitted to GenBank with the accession number MT784750.

The complete chloroplast genome of *M. pterleifolia* (MT784750) is 159,012 bp in length, separated by a small single-copy (SSC) region (18,609 bp) and a large single-copy (LSC) region (85,123 bp), consisting of a pair of inverted repeats (IRs: 27,640 bp each). The overall GC content of the cp genome is 38.33%. There are 131 genes reported, including 84 protein-coding genes, 8 ribosomal RNA genes, 37 tRNA genes, and 2 pseudogenes.

For phylogenetic analysis, a maximum likelihood (ML) tree was constructed by the complete chloroplast genome sequences of *M. pterleifolia* and 17 species from Rutaceae family, with *Dimocarpus longan* as an outgroup. All of the sequences were downloaded from NCBI GenBank. The ML analysis was constructed by RAxML software (Stamatakis 2014) with 1000 bootstrap replicates. The ML tree (Figure 1) showed that *M. pterleifolia* was clustered together with other species from Rutoideae and *Casimiroa edulis* from Toddalioideae. This finding could serve as a valuable genomic resource for genetic researches on Rutaceae in the future.

**Figure 1.**
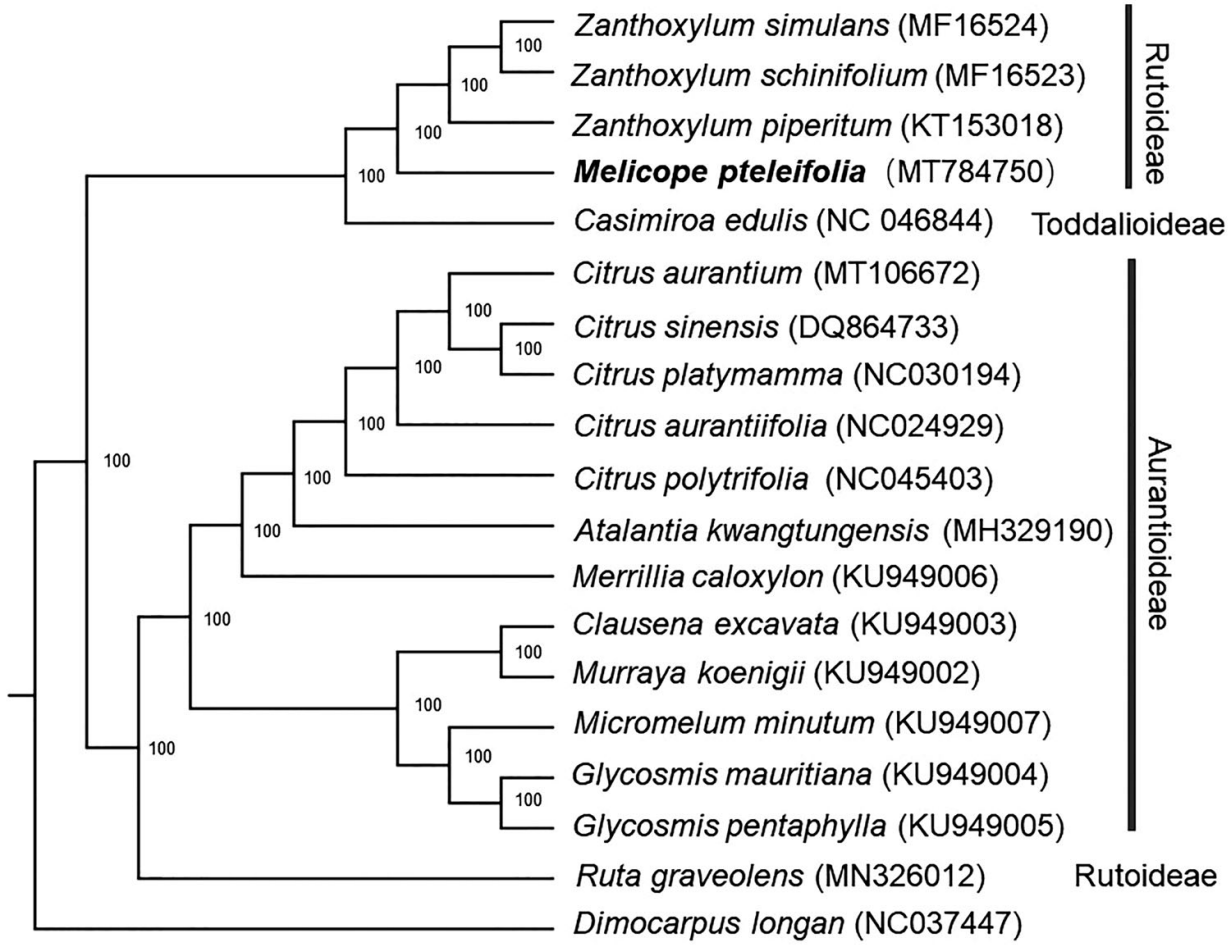
The maximum-likelihood (ML) analysis of 15 species of *Rutaceae* with *Dimocarpus longan* as outgroup based on chloroplast genome sequences. Numbers near the nodes are bootstrap support values.

## Data Availability

The data that support the findings of this study are openly available in NCBI at https://www.ncbi.nlm.nih.gov/, reference number MT784750.
